# Lung Cancer Incidence in Nonmetropolitan and Metropolitan Counties — United States, 2007–2016

**DOI:** 10.15585/mmwr.mm6844a1

**Published:** 2019-11-08

**Authors:** Mary Elizabeth O’Neil, S. Jane Henley, Elizabeth A. Rohan, Taylor D. Ellington, M. Shayne Gallaway

**Affiliations:** 1 Division of Cancer Prevention and Control, National Center for Chronic Disease Prevention and Health Promotion, CDC.

Lung and bronchus (lung) cancer is the leading cause of cancer death in the United States (*1*). In 2016, 148,869 lung cancer deaths were reported.* Most lung cancers can be attributed to modifiable exposures, such as tobacco use, secondhand smoke, radon, and asbestos (*1*). Exposure to lung cancer risk factors vary over time and by characteristics such as sex, age, and nonmetropolitan or metropolitan residence that might affect lung cancer rates (*1*,*2*). A recent report found that lung cancer incidence rates were higher and decreased more slowly in nonmetropolitan counties than in metropolitan counties (*3*). To examine whether lung cancer incidence trends among nonmetropolitan and metropolitan counties differed by age and sex, CDC analyzed data from U.S. Cancer Statistics during 2007–2016, the most recent years for which data are available. During the 10-year study period, lung cancer incidence rates were stable among females aged <35, 45–64, and ≥75 years in nonmetropolitan counties, were stable among females aged <35 years in metropolitan counties, and decreased in all other groups. Overall, among males, lung cancer incidence rates decreased from 99 to 82 per 100,000 in nonmetropolitan areas and from 83 to 63 in metropolitan areas; among females, lung cancer incidence rates decreased from 61 to 58 in nonmetropolitan areas and from 57 to 50 in metropolitan areas. A comprehensive approach to lung cancer prevention and control includes such population-based strategies as screening for tobacco dependence, promoting tobacco cessation, implementing comprehensive smoke-free laws, testing all homes for radon and using proven methods to lower high radon levels, and reducing exposure to lung carcinogens such as asbestos (*1*). Increasing the implementation of these strategies, particularly among persons living in nonmetropolitan counties, might help to reduce disparities in the decline of lung cancer incidence.

Data on new cases of invasive lung cancers^†^ diagnosed during 2007–2016 were obtained from U.S. Cancer Statistics. During this 10-year period, data from all registries met data quality criteria,^§^ but county-level data were not available for Kansas and Minnesota; therefore, data in this report cover approximately 97% of the U.S. population. The U.S. Department of Agriculture Economic Research Service 2013 vintage rural-urban continuum classification scheme was used to categorize county of residence at diagnosis as nonmetropolitan (rural-urban continuum codes 4–9) or metropolitan (rural-urban continuum codes 1–3).^¶^

Calculation of annual incidence rates per 100,000 persons used modified annual population estimates in the denominator and was age-adjusted by the direct method to the 2000 U.S. standard population.** Rates were examined by sex, age group, and nonmetropolitan or metropolitan county status. Rate ratios were calculated to test whether sex-, age- and year-specific rates in nonmetropolitan counties differed from those in metropolitan counties; rates were considered significantly different (p<0.05) if the 95% confidence interval (CI) for the rate ratio excluded one. Annual percentage change (APC) was used to quantify the change in incidence over time and was calculated using least-squares regression. A two-sided t-test was used to determine whether APC was significantly different from zero. Rates were considered to increase if APC >0 (p<0.05) and to decrease if APC <0 (p<0.05); otherwise rates were considered stable. Absolute change was calculated as the difference in incidence from 2007 to 2016. To allow for informal comparisons, without specifying a referent group, 95% CIs for rates and APCs are presented. Analyses were performed using SEER*Stat software (version 8.3.6; National Cancer Institute).

From 2007 to 2016, lung cancer incidence rates declined in both nonmetropolitan and metropolitan counties among both males and females, but the rate of decline differed by sex and rural-urban status. In 2007, lung cancer incidence rates among males in nonmetropolitan counties (99 per 100,000) were 60% higher than that among females in nonmetropolitan counties (61 per 100,000); in 2016, the rate among males (82 per 100,000) in nonmetropolitan counties was 40% higher than that of females in nonmetropolitan counties (58 per 100,000) (Figure 1).

**FIGURE 1:**
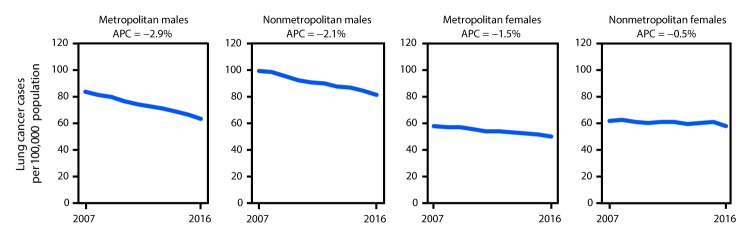
Trends* in lung cancer incidence rates^†^ in nonmetropolitan and metropolitan counties,^§^ by sex — United States,^¶^ 2007–2016 **Abbreviation:** APC = annual percentage change. * Trends were measured with APC in rates; all APCs were significantly different from zero (p<0.05). ^†^ Per 100,000 persons and age-adjusted to the 2000 U.S. standard population. ^§^ The U.S. Department of Agriculture Economic Research Service 2013 vintage rural-urban continuum codes were used to categorize county residence at time of cancer diagnosis as nonmetropolitan (codes 4–9) or metropolitan (codes 1–3). https://www.ers.usda.gov/data-products/rural-urban-continuum-codes. ^¶^ Cancer incidence data were compiled from 49 cancer registries that meet the data quality criteria for all invasive cancer sites combined, representing approximately 97% of the U.S. population. (County-level data were not available for Kansas and Minnesota.)

In metropolitan areas, incidence rates declined more sharply among both males (APC = −2.9%) and females (−1.5%) than it did among males (−2.1%) and females (−0.5%) in nonmetropolitan areas (Figure 1). Lung cancer incidence rates decreased among males in all age groups in both nonmetropolitan and metropolitan counties. Among males, the largest declines were among those aged 45–54 years in metropolitan counties (APC = −5.2%) and those aged 35–44 years in nonmetropolitan counties (APC = −5.0%) (Table). Lung cancer incidence rates also decreased among females in metropolitan counties for most age groups, except those aged <35 years; the largest decline was among females aged 35–44 years in metropolitan counties (APC = −5.0%). Among females in nonmetropolitan counties, incidence rates declined among those aged 35–44 years (APC = −3.6%) and 65–74 years (APC = −1.3%) and were stable in all other age groups (Table).

**TABLE:** Number and rate* of lung cancer cases, absolute rate change, and annual percentage change (APC) in rates in nonmetropolitan and metropolitan counties^†^ by sex and age at diagnosis — United States, ^§^ 2007–2016

Sex, county status, age group (yrs)	2007			2016			Change in rate 2007–2016	
	No.	Rate (95% CI)	RR	No.	Rate (95% CI)	RR	Absolute rate change	APC
**Males**								
**Metropolitan total**	**91,100**	**83.1 (82.6 to 83.7)**	**1.00**	**89,260**	**63 (62.6 to 63.4)**	**1.00**	**−20.2**	−**2.9 (**−**3.2 to** −**2.7)**^¶^
<35	215	0.4 (0.3 to 0.4)	1.00	226	0.3 (0.3 to 0.4)	1.00	0.0	−1.6 (−3.2 to −0.1)^¶^
35–44	1,261	7.0 (6.7 to 7.4)	1.00	749	4.4 (4.1 to 4.8)	1.00	−2.6	−4.8 (−6.1 to −3.5)^¶^
45–54	8,310	46.5 (45.5 to 47.5)	1.00	5,239	28.4 (27.6 to 29.2)	1.00	−18.1	−5.2 (−5.8 to −4.5)^¶^
55–64	20,371	159.1 (156.9 to 161.3)	1.00	20,914	126.4 (124.7 to 128.2)	1.00	−32.6	−2.4 (−2.9 to −2.0)^¶^
65–74	28,977	410.8 (406.0 to 415.6)	1.00	31,887	304.7 (301.4 to 308.1)	1.00	−106.1	−3.2 (−3.4 to −3.0)^¶^
≥75	31,966	572.4 (566.1 to 578.7)	1.00	30,245	449.1 (444.0 to 454.2)	1.00	−123.3	−2.5 (−2.8 to −2.2)^¶^
**Nonmetropolitan total**	**24,166**	**99.0 (97.7 to 100.3)**	1.19**	**23,712**	**81.5 (80.5 to 82.6)**	1.29**	−**17.4**	−**2.1 (**−**2.3 to** −**1.9)**^¶^
<35	46	0.5 (0.4 to 0.7)	1.37	26	0.3 (0.2 to 0.4)	0.80	−0.2	−3.9 (−6.8 to −0.9)^¶^
35–44	283	9.6 (8.5 to 10.8)	1.36**	163	6.5 (5.5 to 7.5)	1.46**	−3.1	−5.0 (−6.4 to −3.6)^¶^
45–54	2,058	61.5 (58.9 to 64.3)	1.32**	1,428	47.3 (44.9 to 49.9)	1.67**	−14.2	−2.8 (−3.6 to −1.9)^¶^
55–64	5,562	205.4 (200.1 to 210.9)	1.29**	5,657	182.1 (177.4 to 186.9)	1.44**	−23.3	−1.1 (−1.6 to −0.7)^¶^
65–74	8,395	496.3 (485.7 to 507.1)	1.21**	8,810	396.7 (388.4 to 405.2)	1.30**	−99.6	−2.5 (−2.7 to −2.2)^¶^
≥75	7,822	632.6 (618.6 to 646.8)	1.11**	7,628	528.5 (516.7 to 540.5)	1.18**	−104.1	−1.9 (−2.1 to −1.7)^¶^
**Females**								
**Metropolitan total**	**80,316**	**57.3 (56.9 to 57.7)**	**1.00**	**86,220**	**49.7 (49.3 to 50)**	**1.00**	−**7.6**	−**1.5 (**−**1.7 to** −**1.3)**^¶^
<35	216	0.4 (0.3 to 0.4)	1.00	226	0.3 (0.3 to 0.4)	1.00	0.0	−1.2 (−2.9 to 0.5)
35–44	1,343	7.4 (7.0 to 7.8)	1.00	832	4.8 (4.5 to 5.2)	1.00	−2.5	−5.0 (−5.9 to −4.2)^¶^
45–54	7,495	40.2 (39.3 to 41.1)	1.00	5,756	30.2 (29.4 to 31.0)	1.00	−10.0	−3.0 (−3.8 to −2.1)^¶^
55–64	16,489	118.2 (116.4 to 120.0)	1.00	19,150	106.9 (105.4 to 108.4)	1.00	−11.3	−0.9 (−1.7 to −0.1)^¶^
65–74	24,723	294.7 (291.1 to 298.4)	1.00	29,402	242.4 (239.7 to 245.3)	1.00	−52.3	−2.1 (−2.3 to −1.8)^¶^
≥75	30,050	343.4 (339.5 to 347.3)	1.00	30,854	320.2 (316.6 to 323.8)	1.00	−23.2	−0.8 (−1.2 to −0.5)^¶^
**Nonmetropolitan total**	**17,694**	**61.2 (60.3 to 62.2)**	1.07**	**18,920**	**57.9 (57.1 to 58.8)**	1.17**	−**3.3**	−**0.5 (**−**0.8 to** −**0.2)**^¶^
<35	33	0.4 (0.3 to 0.5)	1.05	36	0.4 (0.3 to 0.6)	1.18	0.0	−0.6 (−5.2 to 4.2)
35–44	317	11.0 (9.9 to 12.3)	1.50**	187	7.8 (6.7 to 9.0)	1.61**	−3.3	−3.6 (−5.1 to −2.2)^¶^
45–54	1,712	51.9 (49.5 to 54.4)	1.29**	1,490	50.1 (47.6 to 52.7)	1.66**	−1.8	−0.6 (−1.6 to 0.5)
55–64	3,788	136.5 (132.2 to 141.0)	1.16**	4,584	142.8 (138.7 to 147.1)	1.34**	6.3	0.7 (−0.2 to 1.6)
65–74	5,962	320.3 (312.2 to 328.5)	1.09**	6,673	280.5 (273.8 to 287.4)	1.16**	−39.8	−1.3 (−1.7 to −0.9)^¶^
≥75	5,882	318.0 (309.9 to 326.3)	0.93**	5,950	309.9 (302.0 to 317.9)	0.97**	−8.2	0.0 (−0.5 to 0.4)

**Abbreviations:** CI = confidence interval; RR = rate ratio.

* Per 100,000 persons; overall rates were age-adjusted to the 2000 U.S. standard population.

^†^ The U.S. Department of Agriculture Economic Research Service 2013 vintage rural-urban continuum codes were used to categorize county residence at time of cancer diagnosis as nonmetropolitan (codes 4–9) or metropolitan (codes 1–3). https://www.ers.usda.gov/data-products/rural-urban-continuum-codes.

^§^ Cancer incidence data were compiled from 49 cancer registries that meet the data quality criteria for all invasive cancer sites combined, representing approximately 97% of the U.S. population. (County-level data were not available for Kansas and Minnesota.)

^¶^ APC was significantly different from zero at p<0.05. Trends were measured with APC in rates and were considered to increase or decrease if p<0.05; otherwise rates were considered stable.

** Sex-, age-, and year-specific rates in nonmetropolitan counties were significantly different from rates in metropolitan counties.

In 2016, among persons aged ≥55 years, the highest lung cancer incidence rates were observed among men in nonmetropolitan counties (Figure 2). Among persons aged 35–54 years, rates in nonmetropolitan and metropolitan counties did not differ by sex but were higher in nonmetropolitan counties than in metropolitan counties. Rates were higher among women aged 35–64 years in nonmetropolitan counties than among men in metropolitan counties (Figure 2).

**FIGURE 2:**
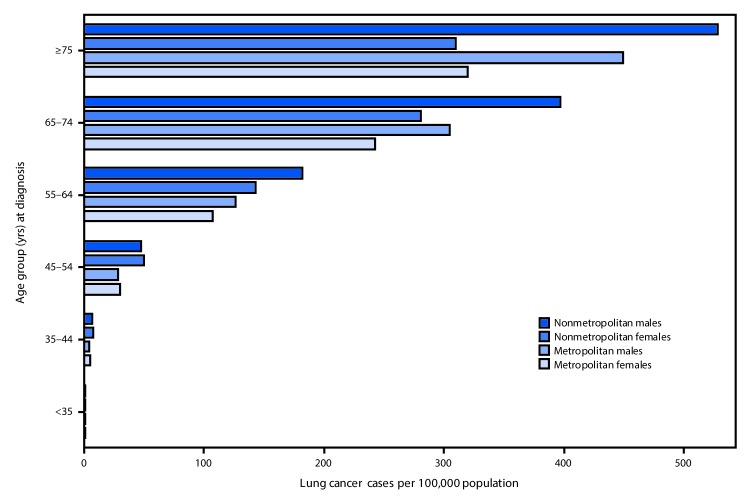
Rate* of lung cancer in nonmetropolitan and metropolitan counties,^†^ by sex and age at diagnosis — United States,^§^ 2016 * Per 100,000 persons and age-adjusted to the 2000 U.S. standard population. ^†^ The U.S. Department of Agriculture Economic Research Service 2013 vintage rural-urban continuum codes were used to categorize county residence at time of cancer diagnosis as nonmetropolitan (codes 4–9) or metropolitan (codes 1–3). https://www.ers.usda.gov/data-products/rural-urban-continuum-codes. ^§^ Cancer incidence data were compiled from 49 cancer registries that meet the data quality criteria for all invasive cancer sites combined, representing approximately 97% of the U.S. population. (County-level data were not available for Kansas and Minnesota.)

## Discussion

Although lung cancer incidence rates declined among males and females living in nonmetropolitan and metropolitan areas during 2007–2016, the smallest decrease occurred among females living in nonmetropolitan counties, who also experienced high incidence in some age groups. During this 10-year period, the highest overall lung cancer incidence rates were observed among males in nonmetropolitan counties. National Health Interview Survey 2017 data indicate that, compared with adults living in metropolitan areas, those living in nonmetropolitan areas reported a higher prevalence of current cigarette smoking (23% versus 13%) and a lower prevalence of quit attempts (50% versus 56%) and successful cessation (5% versus 9%) (*4*).

Lung cancer prevention and control is a comprehensive approach and includes strategies such as screening for tobacco dependence, promoting tobacco cessation, implementing comprehensive smoke-free laws, testing all homes for radon and using proven methods to lower high radon levels, and reducing exposure to lung carcinogens such as asbestos (*1*). The U.S. Preventive Services Task Force recommends that clinicians screen all adults for tobacco use at each office visit and refer or provide behavioral and pharmacotherapy smoking cessation interventions as indicated.^††^ Lung cancer screening is recommended for adults at high risk for developing lung cancer because of their age and cigarette smoking history. Screening efforts can identify lung cancer in its early stages and provide an important opportunity to promote tobacco smoking cessation. However, access to these preventive services might be more limited in nonmetropolitan areas, where a higher percentage of residents aged <65 years report being uninsured compared with those in metropolitan areas (*4*).

CDC’s National Comprehensive Cancer Control Program^§§^ funds state, tribal, local, and territorial comprehensive cancer control programs that pool resources to lower the number of persons affected by types of cancer with the highest burden in a given community, including lung cancer. These programs advance their priorities through evidence-based interventions that include primary prevention and early detection. Examples of lung cancer prevention strategies are promoting tobacco-free living for all persons (*5*) and reducing exposure to indoor radon (*6*). An important step in implementing interventions for the early detection of lung cancer is assessing a community’s capacity to meet screening needs. For example, Maine’s Comprehensive Cancer Control Program identified lung cancer screening facilities in nonmetropolitan and metropolitan areas and is working to address screening barriers (*7*). Another approach is using patient navigators and community health workers to address health care barriers (e.g., financial hardships, lack of or inadequate health insurance coverage, and lack of transportation) (*8*). CDC, along with the Appalachian Regional Commission, has funded research to more fully understand how patient navigation can help cancer survivors in nonmetropolitan areas have better access to cancer care,^¶¶^ which can then inform the development of culturally relevant training for patient navigators.

Although cigarette smoking is the primary cause of lung cancer, other risk factors, which may differ by geographic region, include use of other smoking tobacco products and exposure to secondhand smoke, indoor radon, and asbestos (*1*). In some states, rural areas may be less likely to have strong smoke-free laws or barrier-free access to tobacco cessation programs.***

Approximately 10%–15% of lung cancers are estimated to occur among persons who have never smoked cigarettes (*9*). Regardless of smoking status, lung cancer survivors might experience blame, stigma, and other negative reactions associated with their lung cancer diagnosis (*10*). A qualitative analysis found that lung cancer survivors believed the stigma translated into a lack of public empathy, and they desired increased public support (*10*). Public health programs such as CDC’s National Comprehensive Cancer Control Program are focused on cancer survivorship and can work to reduce stigma by educating the public and implementing programs to address the needs of lung cancer survivors.

The findings in this report are subject to at least two limitations. First, delays in cancer reporting might result in an underestimation of incidence. Second, incidence was not determinable by county classification for all states; therefore, these results might not apply to states excluded from the analyses.

During 2007–2016, lung cancer incidence rates declined overall in nonmetropolitan and metropolitan counties; however, rates decreased more in metropolitan than in nonmetropolitan counties, more among males than among females, and more among persons aged 35–54 years than among those aged ≥55 years. As a result, differences in lung cancer incidence rates between males and females narrowed with decreasing age, but disparities by rural-urban status persisted. A comprehensive approach to lung cancer prevention and control includes such population-based strategies as screening for tobacco dependence, promoting tobacco cessation, implementing comprehensive smoke-free laws, testing all homes for radon and using proven methods to lower high radon levels, and reducing exposure to lung carcinogens such as asbestos (*1*). Increasing the implementation of proven population-based lung cancer prevention and control strategies, particularly among persons living in nonmetropolitan areas, might help to reduce disparities in the decline of lung cancer incidence.

**Summary**

**What is already known about this topic?**

Preventing cigarette smoking and exposure to secondhand smoke, radon, and asbestos might reduce lung cancer risk. Exposure to some risk factors might vary by characteristics such as sex, age, and urban or rural residence, which might affect the occurrence of new lung cancers.

**What is added by this report?**

During 2007–2016, lung cancer incidence rates decreased more in metropolitan than nonmetropolitan counties, more among males than females, and more among middle-aged adults than older adults.

**What are the implications for public health practice?**

Accelerating implementation of proven strategies to reduce exposure to lung cancer risk factors, particularly among females living in nonmetropolitan areas, might prevent lung cancer and decrease disparities.

## Footnotes

* https://www.cdc.gov/cancer/uscs.

† http://www.iacr.com.fr/index.php?option=com_content&view=category&layout=blog&id=100&Itemid=577.

§ https://www.cdc.gov/cancer/uscs/technical_notes/criteria/index.htm.

¶ https://www.ers.usda.gov/data-products/rural-urban-continuum-codes.

** https://seer.cancer.gov/popdata.

†† https://www.uspreventiveservicestaskforce.org/Page/Name/recommendations.

§§ https://www.cdc.gov/cancer/ncccp/index.htm.

¶¶ https://www.cecentral.com/node/1466.

*** https://www.cdc.gov/tobacco/disparities/geographic/index.htm.
